# Crystal structure of quinolinium 2-carboxy-6-nitro­benzoate monohydrate

**DOI:** 10.1107/S2056989015006052

**Published:** 2015-04-02

**Authors:** J. Mohana, M. Divya Bharathi, G. Ahila, G. Chakkaravarthi, G. Anbalagan

**Affiliations:** a Department of Physics, Presidency College, Chennai 600 005, India; b Department of Physics, CPCL Polytechnic College, Chennai 600 068, India

**Keywords:** crystal structure, mol­ecular salt, quinolinium, 2-carboxy-6-nitro­benzoate, hydrogen bonding, π–π stacking inter­actions

## Abstract

In the anion of the title hydrated mol­ecular salt, C_9_H_8_N^+^·C_8_H_4_NO_6_^−^·H_2_O, the protonated carboxyl and nitro groups makes dihedral angles of 27.56 (5) and 6.86 (8)°, respectively, with the attached benzene ring, whereas the deprotonated carb­oxy group is almost orthogonal to it with a dihedral angle of 80.21 (1)°. In the crystal, the components are linked by O—H⋯O and N—H⋯O hydrogen bonds, generating [001] chains. The packing is consolidated by weak C—H⋯N and C—H⋯O inter­actions as well as aromatic π–π stacking [centroid-to-centroid distances: 3.7023 (8) & 3.6590 (9)Å] inter­actions, resulting in a three-dimensional network.

## Related literature   

For the biological activity of quinoline derivatives, see: Font *et al.* (1997 ▸); Sloboda *et al.* (1991 ▸). For similar structures, see: Castañeda *et al.* (2014 ▸); Kafka *et al.* (2012 ▸); Li & Chai (2007 ▸); Divya Bharathi *et al.* (2015 ▸).
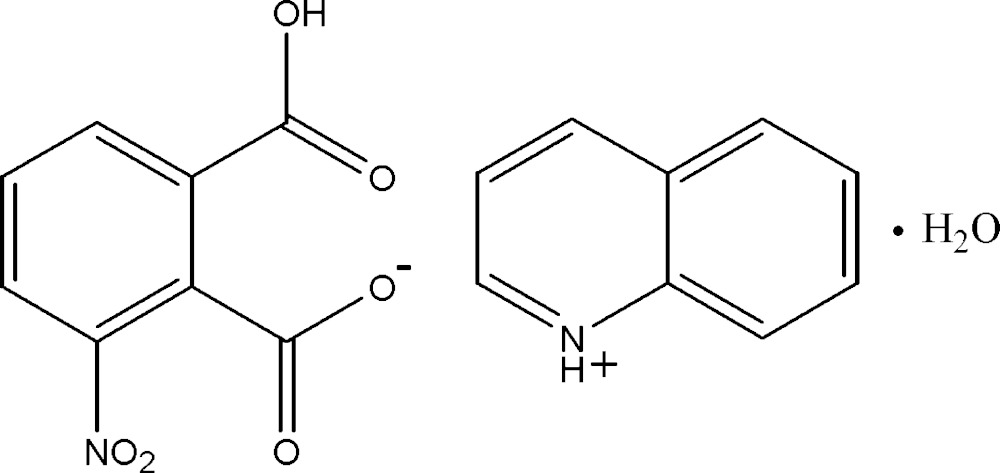


## Experimental   

### Crystal data  

C_9_H_8_N^+^·C_8_H_4_NO_6_^−^·H_2_O
*M* *_r_* = 358.30
Monoclinic, 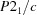

*a* = 14.7622 (8) Å
*b* = 14.2461 (8) Å
*c* = 7.6395 (4) Å
β = 104.434 (2)°
*V* = 1555.90 (15) Å^3^
*Z* = 4
Mo *K*α radiation
μ = 0.12 mm^−1^
*T* = 295 K
0.26 × 0.24 × 0.18 mm

### Data collection  

Bruker Kappa APEXII CCD diffractometer
Absorption correction: multi-scan (*SADABS*; Bruker, 2004 ▸) *T*_min_ = 0.969, *T*_max_ = 0.979
32650 measured reflections
4728 independent reflections
3394 reflections with *I* > 2σ(*I*)
*R*_int_ = 0.025

### Refinement  

*R*[*F* ^2^ > 2σ(*F* ^2^)] = 0.039
*wR*(*F* ^2^) = 0.115
*S* = 1.04
4728 reflections
248 parameters
4 restraints
H atoms treated by a mixture of independent and constrained refinement
Δρ_max_ = 0.29 e Å^−3^
Δρ_min_ = −0.20 e Å^−3^

### 

Data collection: *APEX2* (Bruker, 2004 ▸); cell refinement: *SAINT* (Bruker, 2004 ▸); data reduction: *SAINT*; program(s) used to solve structure: *SHELXS97* (Sheldrick, 2008 ▸); program(s) used to refine structure: *SHELXL97* (Sheldrick, 2008 ▸); molecular graphics: *PLATON* (Spek, 2009 ▸); software used to prepare material for publication: *SHELXL97*.

## Supplementary Material

Crystal structure: contains datablock(s) global, I. DOI: 10.1107/S2056989015006052/hb7390sup1.cif

Structure factors: contains datablock(s) I. DOI: 10.1107/S2056989015006052/hb7390Isup2.hkl

Supporting information file. DOI: 10.1107/S2056989015006052/hb7390Isup3.cml

**Fig. 1.**
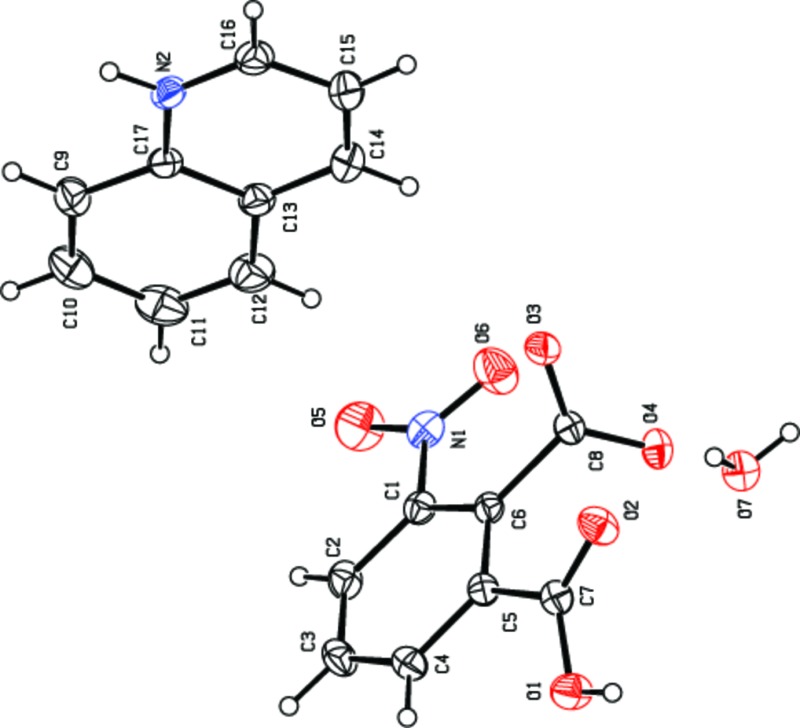
The molecular structure of (I), with 30% probability displacement ellipsoids for non-H atoms.

. DOI: 10.1107/S2056989015006052/hb7390fig1.tif
The mol­ecular structure of (I), with 30% probability displacement ellipsoids for non-H atoms.

**Fig. 2.**
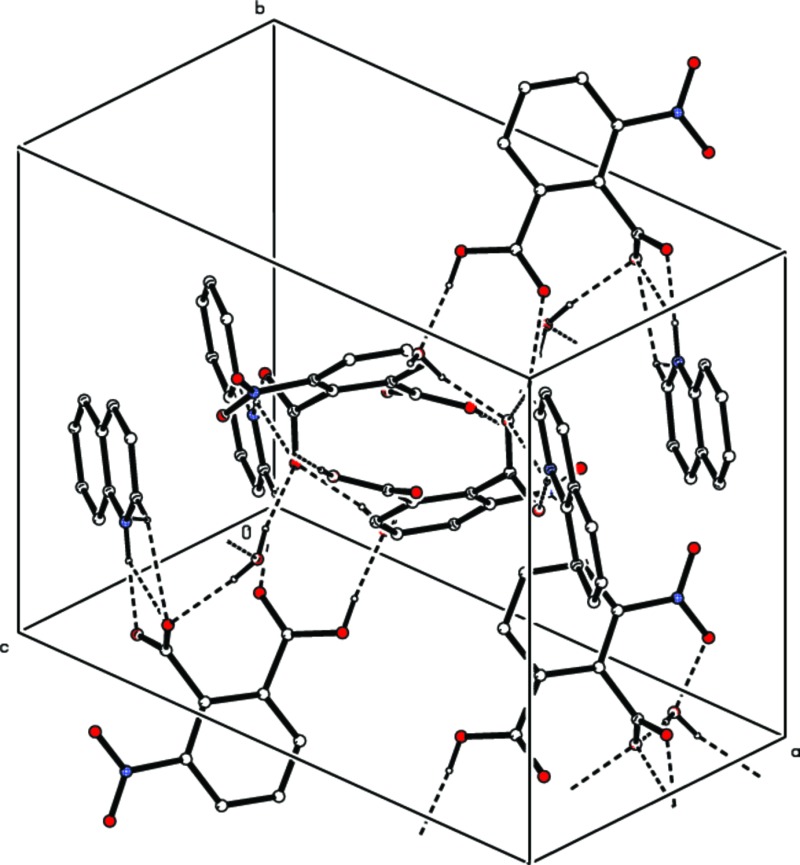
The packing of (I), viewed down C face. Intermolecular Hydrogen bonds are shown as dashed lines. H atoms not involved in hydrogen bonding have been omitted.

. DOI: 10.1107/S2056989015006052/hb7390fig2.tif
The packing of (I), viewed down C face. Inter­molecular Hydrogen bonds are shown as dashed lines. H atoms not involved in hydrogen bonding have been omitted.

CCDC reference: 1015219

Additional supporting information:  crystallographic information; 3D view; checkCIF report

## Figures and Tables

**Table 1:** Hydrogen-bond geometry (, )

*D*H*A*	*D*H	H*A*	*D* *A*	*D*H*A*
O7H7*A*O4	0.83(1)	1.92(1)	2.7416(14)	171(19)
O7H7*B*O4^i^	0.84(1)	2.02(1)	2.8459(14)	169(18)
O1H1*A*O7^ii^	0.84(1)	1.75(1)	2.5818(14)	173(2)
N2H2*A*O3^iii^	0.91(1)	1.74(1)	2.6425(14)	176(18)
C16H16O4^iii^	0.93	2.49	3.1166(17)	125
C16H16O2^iv^	0.93	2.39	3.1278(17)	136
C12H12N1	0.93	2.61	3.4866(19)	157

Symmetry codes: (i) 
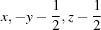
; (ii) 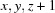; (iii) 
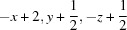
; (iv) 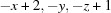.

## supplementary crystallographic information

### S1. Chemical context

The quinoline derivatives are known to exhibit wide range of pharmacological activities such as anti­bacterial (Benzerka, *et al.*2012), anti-viral (Font *et al.*, 1997) and anti-inflammatory (Sloboda *et al.*, 1991). In view of the above importance, we have synthesized the title compound and report herein on its crystal structure.

### S2. Structural commentary

The ORTEP diagram of the title compound (I) is shown in Fig.1. The asymmetric unit of the title compound consists of C_9_ H_8_ N^+^ cation, C_8_ H_4_ N O_6_^-^ anion and a water molecule. The geometric parameters of (I) (Fig. 1) are well agreed with the similar reported structures [Castañeda *et al.*, 2014; Kafka *et al.*, 2012; Li & Chai (2007)].

The quinolinium ring system is planar [r.m.s deviation = 0.0133 (12)Å] and protonated at N2 atom. In the anion, the carboxyl (O1/C7/O2), nitro (O5/N1/O6) and carb­oxy (O3/C8/O4) groups are inclined at an angle of 27.56 (5), 6.86 (8) and 80.21 (1)°, respectively with the attached benzene ring (C1—C6). The dihedral angle between the quinolinium ring system and benzene ring is 89.91 (5)°.

### S3. Supra­molecular features

The molecular structure is stabilized by weak intra­molecular O—H···O and C—H···N hydrogen bonds (Table 1). The crystal structure is stabilized by weak inter­molecular N—H···O, O—H···O and C—H···O hydrogen bonds (Table 1 & Fig. 2) which link adjacent anions and cations through water molecules into infinite one-dimensional chains along [001]. The crystal structure is further stabilized by weak π···π [Cg1···Cg1^i^ = 3.7023 (8); Cg3···Cg2^ii^ = 3.6590 (9)Å; (i) 1-x,-y,-z; (ii) x,1/2-y,-1/2+z; Cg1, Cg2 and Cg3 are the centroids of the rings (C1—C6), (C13/C14/C15/C16/c17/N2) and (C9—C13/c17), respectively] inter­actions.

### S4. Synthesis and crystallization

Quinoline (3.22 g, 0.99 mol) was dissolved in hot water (25 ml) and after half an hour, a solution of 3-nitro­phthalic acid (5.278 g, 0.38 mol) in methanol (25 ml) was added and stirred for about one hour. A white colour powder was formed. The product was dissolved in aqueous methanol solution (210 ml). Single crystals suitable for X-ray diffraction were obtained from the slow evaporation method at room temperature within a few days.

### S5. Refinement

C-bound H atoms were positioned geometrically and refined using riding model with C—H = 0.93 Å and Uiso(H) = 1.2Ueq(C). H atoms for O atoms were located from Fourier map and refined with Uiso(H) = 1.5 Ueq(O). Distance restraints were applied for the distance O—H = 0.82 (1)Å. H atom for N atom was located from Fourier map and refined freely with distance restraint N—H = 0.88 (1)Å.

### Crystal data

**Table:**

C_9_H_8_N^+^·C_8_H_4_NO_6_^−^·H_2_O	*F*(000) = 744
*M**_r_* = 358.30	*D*_x_ = 1.530 Mg m^−^^3^
Monoclinic, *P*2_1_/*c*	Mo *K*α radiation, λ = 0.71073 Å
Hall symbol: -P 2ybc	Cell parameters from 9970 reflections
*a* = 14.7622 (8) Å	θ = 2.8–30.4°
*b* = 14.2461 (8) Å	µ = 0.12 mm^−^^1^
*c* = 7.6395 (4) Å	*T* = 295 K
β = 104.434 (2)°	Block, colourless
*V* = 1555.90 (15) Å^3^	0.26 × 0.24 × 0.18 mm
*Z* = 4	

### Data collection

**Table:**

Bruker Kappa APEXII CCD diffractometer	4728 independent reflections
Radiation source: fine-focus sealed tube	3394 reflections with *I* > 2σ(*I*)
Graphite monochromator	*R*_int_ = 0.025
ω and φ scans	θ_max_ = 30.8°, θ_min_ = 2.9°
Absorption correction: multi-scan (*SADABS*; Bruker, 2004)	*h* = −21→21
*T*_min_ = 0.969, *T*_max_ = 0.979	*k* = −20→20
32650 measured reflections	*l* = −10→9

### Refinement

**Table:**

Refinement on *F*^2^	Primary atom site location: structure-invariant direct methods
Least-squares matrix: full	Secondary atom site location: difference Fourier map
*R*[*F*^2^ > 2σ(*F*^2^)] = 0.039	Hydrogen site location: inferred from neighbouring sites
*wR*(*F*^2^) = 0.115	H atoms treated by a mixture of independent and constrained refinement
*S* = 1.04	*w* = 1/[σ^2^(*F*_o_^2^) + (0.0472*P*)^2^ + 0.4484*P*] where *P* = (*F*_o_^2^ + 2*F*_c_^2^)/3
4728 reflections	(Δ/σ)_max_ < 0.001
248 parameters	Δρ_max_ = 0.29 e Å^−^^3^
4 restraints	Δρ_min_ = −0.20 e Å^−^^3^

### Special details

**Table:**

Refinement. Refinement of F^2^ against ALL reflections. The weighted R-factor wR and goodness of fit S are based on F^2^, conventional R-factors R are based on F, with F set to zero for negative F^2^. The threshold expression of F^2^ > 2sigma(F^2^) is used only for calculating R-factors(gt) etc. and is not relevant to the choice of reflections for refinement. R-factors based on F^2^ are statistically about twice as large as those based on F, and R- factors based on ALL data will be even larger.

### Fractional atomic coordinates and isotropic or equivalent isotropic displacement parameters (Å^2^)

**Table:**

	*x*	*y*	*z*	*U*_iso_*/*U*_eq_	
C1	0.64273 (8)	0.07483 (8)	−0.05905 (16)	0.0309 (2)	
C2	0.57907 (9)	0.14414 (9)	−0.04652 (19)	0.0386 (3)	
H2	0.5629	0.1900	−0.1354	0.046*	
C3	0.54012 (9)	0.14429 (9)	0.09892 (19)	0.0405 (3)	
H3	0.4982	0.1912	0.1106	0.049*	
C4	0.56333 (9)	0.07465 (9)	0.22753 (18)	0.0361 (3)	
H4	0.5351	0.0735	0.3236	0.043*	
C5	0.62851 (8)	0.00608 (8)	0.21529 (16)	0.0295 (2)	
C6	0.67125 (8)	0.00565 (8)	0.07151 (15)	0.0276 (2)	
C7	0.65254 (8)	−0.06752 (8)	0.35872 (16)	0.0321 (2)	
C8	0.74316 (8)	−0.06939 (8)	0.06354 (16)	0.0305 (2)	
C9	0.95815 (10)	0.39076 (10)	0.1010 (2)	0.0450 (3)	
H9	0.9905	0.4451	0.1463	0.054*	
C10	0.87001 (11)	0.39591 (13)	−0.0086 (2)	0.0563 (4)	
H10	0.8421	0.4543	−0.0379	0.068*	
C11	0.82119 (11)	0.31475 (15)	−0.0772 (2)	0.0617 (5)	
H11	0.7610	0.3196	−0.1515	0.074*	
C12	0.86022 (10)	0.22927 (13)	−0.0373 (2)	0.0552 (4)	
H12	0.8268	0.1759	−0.0848	0.066*	
C13	0.95129 (9)	0.22011 (10)	0.07584 (19)	0.0387 (3)	
C14	0.99619 (11)	0.13384 (10)	0.1225 (2)	0.0488 (4)	
H14	0.9653	0.0784	0.0797	0.059*	
C15	1.08465 (11)	0.13063 (10)	0.2302 (2)	0.0497 (4)	
H15	1.1152	0.0734	0.2584	0.060*	
C16	1.12878 (9)	0.21365 (10)	0.2974 (2)	0.0429 (3)	
H16	1.1887	0.2117	0.3734	0.051*	
C17	0.99937 (8)	0.30253 (9)	0.14427 (17)	0.0331 (3)	
N1	0.67819 (8)	0.07495 (8)	−0.22213 (15)	0.0389 (3)	
H2A	1.1177 (11)	0.3480 (9)	0.304 (2)	0.060 (5)*	
N2	1.08727 (7)	0.29511 (8)	0.25544 (16)	0.0364 (2)	
O1	0.58421 (7)	−0.08353 (8)	0.43610 (14)	0.0457 (2)	
H1A	0.6007 (14)	−0.1247 (11)	0.516 (2)	0.069*	
O2	0.72664 (7)	−0.10794 (7)	0.39726 (13)	0.0445 (2)	
O3	0.82733 (6)	−0.04668 (7)	0.11938 (14)	0.0408 (2)	
O4	0.71315 (7)	−0.14892 (6)	0.01135 (13)	0.0386 (2)	
O5	0.65803 (10)	0.14023 (9)	−0.32691 (17)	0.0659 (4)	
O6	0.72700 (9)	0.01019 (9)	−0.24593 (16)	0.0611 (3)	
O7	0.62094 (7)	−0.21451 (7)	−0.32298 (13)	0.0420 (2)	
H7A	0.6537 (12)	−0.1934 (13)	−0.2261 (17)	0.063*	
H7B	0.6482 (12)	−0.2591 (10)	−0.359 (2)	0.063*	

### Atomic displacement parameters (Å^2^)

**Table:**

	*U*^11^	*U*^22^	*U*^33^	*U*^12^	*U*^13^	*U*^23^
C1	0.0293 (5)	0.0308 (6)	0.0300 (6)	−0.0029 (4)	0.0026 (4)	0.0001 (4)
C2	0.0368 (6)	0.0332 (6)	0.0406 (7)	0.0043 (5)	0.0001 (5)	0.0069 (5)
C3	0.0350 (6)	0.0363 (7)	0.0469 (8)	0.0107 (5)	0.0042 (5)	−0.0002 (5)
C4	0.0322 (6)	0.0386 (6)	0.0368 (6)	0.0049 (5)	0.0073 (5)	−0.0027 (5)
C5	0.0273 (5)	0.0287 (5)	0.0296 (6)	0.0008 (4)	0.0014 (4)	−0.0015 (4)
C6	0.0245 (5)	0.0256 (5)	0.0300 (6)	−0.0016 (4)	0.0017 (4)	−0.0027 (4)
C7	0.0341 (6)	0.0325 (6)	0.0281 (6)	0.0010 (5)	0.0047 (5)	−0.0021 (5)
C8	0.0328 (6)	0.0293 (5)	0.0290 (6)	0.0023 (4)	0.0068 (4)	−0.0014 (4)
C9	0.0443 (7)	0.0386 (7)	0.0511 (8)	0.0053 (6)	0.0100 (6)	0.0017 (6)
C10	0.0489 (9)	0.0593 (10)	0.0580 (10)	0.0201 (7)	0.0085 (7)	0.0066 (8)
C11	0.0353 (7)	0.0846 (13)	0.0584 (10)	0.0099 (8)	−0.0011 (7)	−0.0028 (9)
C12	0.0359 (7)	0.0647 (10)	0.0604 (10)	−0.0080 (7)	0.0031 (7)	−0.0138 (8)
C13	0.0316 (6)	0.0414 (7)	0.0437 (7)	−0.0041 (5)	0.0105 (5)	−0.0058 (6)
C14	0.0479 (8)	0.0350 (7)	0.0644 (10)	−0.0077 (6)	0.0157 (7)	−0.0050 (6)
C15	0.0469 (8)	0.0350 (7)	0.0684 (10)	0.0049 (6)	0.0166 (7)	0.0093 (7)
C16	0.0311 (6)	0.0444 (7)	0.0519 (8)	0.0019 (5)	0.0080 (6)	0.0107 (6)
C17	0.0282 (5)	0.0364 (6)	0.0355 (6)	−0.0006 (5)	0.0094 (5)	0.0014 (5)
N1	0.0401 (6)	0.0412 (6)	0.0338 (6)	−0.0060 (5)	0.0061 (5)	0.0015 (5)
N2	0.0289 (5)	0.0362 (5)	0.0429 (6)	−0.0049 (4)	0.0068 (4)	0.0021 (5)
O1	0.0425 (5)	0.0521 (6)	0.0446 (6)	0.0056 (4)	0.0148 (4)	0.0143 (4)
O2	0.0439 (5)	0.0486 (6)	0.0404 (5)	0.0147 (4)	0.0095 (4)	0.0113 (4)
O3	0.0286 (4)	0.0385 (5)	0.0539 (6)	0.0020 (4)	0.0078 (4)	−0.0039 (4)
O4	0.0459 (5)	0.0293 (4)	0.0388 (5)	0.0001 (4)	0.0071 (4)	−0.0059 (4)
O5	0.0789 (9)	0.0694 (8)	0.0530 (7)	0.0109 (6)	0.0232 (6)	0.0290 (6)
O6	0.0824 (9)	0.0594 (7)	0.0506 (6)	0.0141 (6)	0.0338 (6)	0.0025 (5)
O7	0.0478 (6)	0.0417 (5)	0.0355 (5)	0.0014 (4)	0.0086 (4)	0.0010 (4)

### Geometric parameters (Å, º)

**Table:**

C1—C2	1.3828 (17)	C10—H10	0.9300
C1—C6	1.3907 (16)	C11—C12	1.349 (3)
C1—N1	1.4666 (16)	C11—H11	0.9300
C2—C3	1.372 (2)	C12—C13	1.4114 (19)
C2—H2	0.9300	C12—H12	0.9300
C3—C4	1.3780 (19)	C13—C14	1.400 (2)
C3—H3	0.9300	C13—C17	1.4039 (18)
C4—C5	1.3906 (16)	C14—C15	1.360 (2)
C4—H4	0.9300	C14—H14	0.9300
C5—C6	1.3965 (17)	C15—C16	1.386 (2)
C5—C7	1.4940 (16)	C15—H15	0.9300
C6—C8	1.5184 (16)	C16—N2	1.3141 (17)
C7—O2	1.2060 (15)	C16—H16	0.9300
C7—O1	1.3101 (16)	C17—N2	1.3663 (16)
C8—O4	1.2453 (14)	N1—O6	1.2120 (16)
C8—O3	1.2516 (14)	N1—O5	1.2146 (15)
C9—C10	1.362 (2)	N2—H2A	0.908 (9)
C9—C17	1.3996 (19)	O1—H1A	0.840 (9)
C9—H9	0.9300	O7—H7A	0.833 (9)
C10—C11	1.394 (3)	O7—H7B	0.836 (9)
			
C2—C1—C6	123.08 (12)	C12—C11—C10	120.79 (14)
C2—C1—N1	116.78 (11)	C12—C11—H11	119.6
C6—C1—N1	120.12 (11)	C10—C11—H11	119.6
C3—C2—C1	119.09 (12)	C11—C12—C13	120.66 (15)
C3—C2—H2	120.5	C11—C12—H12	119.7
C1—C2—H2	120.5	C13—C12—H12	119.7
C2—C3—C4	119.75 (12)	C14—C13—C17	118.44 (12)
C2—C3—H3	120.1	C14—C13—C12	123.75 (13)
C4—C3—H3	120.1	C17—C13—C12	117.82 (13)
C3—C4—C5	120.73 (12)	C15—C14—C13	120.41 (13)
C3—C4—H4	119.6	C15—C14—H14	119.8
C5—C4—H4	119.6	C13—C14—H14	119.8
C4—C5—C6	120.78 (11)	C14—C15—C16	119.16 (13)
C4—C5—C7	119.05 (11)	C14—C15—H15	120.4
C6—C5—C7	120.17 (10)	C16—C15—H15	120.4
C1—C6—C5	116.47 (10)	N2—C16—C15	121.06 (12)
C1—C6—C8	124.05 (11)	N2—C16—H16	119.5
C5—C6—C8	119.47 (10)	C15—C16—H16	119.5
O2—C7—O1	124.07 (12)	N2—C17—C9	120.40 (12)
O2—C7—C5	123.32 (11)	N2—C17—C13	118.71 (11)
O1—C7—C5	112.60 (10)	C9—C17—C13	120.89 (12)
O4—C8—O3	126.02 (11)	O6—N1—O5	122.75 (13)
O4—C8—C6	117.19 (10)	O6—N1—C1	118.55 (11)
O3—C8—C6	116.67 (10)	O5—N1—C1	118.69 (12)
C10—C9—C17	119.08 (14)	C16—N2—C17	122.19 (11)
C10—C9—H9	120.5	C16—N2—H2A	118.7 (12)
C17—C9—H9	120.5	C17—N2—H2A	119.1 (12)
C9—C10—C11	120.77 (15)	C7—O1—H1A	109.7 (14)
C9—C10—H10	119.6	H7A—O7—H7B	110.4 (18)
C11—C10—H10	119.6		
			
C6—C1—C2—C3	−1.47 (19)	C17—C9—C10—C11	−0.1 (3)
N1—C1—C2—C3	176.70 (11)	C9—C10—C11—C12	−0.2 (3)
C1—C2—C3—C4	−1.4 (2)	C10—C11—C12—C13	0.3 (3)
C2—C3—C4—C5	2.4 (2)	C11—C12—C13—C14	−179.82 (16)
C3—C4—C5—C6	−0.64 (18)	C11—C12—C13—C17	−0.1 (2)
C3—C4—C5—C7	179.28 (12)	C17—C13—C14—C15	−0.7 (2)
C2—C1—C6—C5	3.15 (17)	C12—C13—C14—C15	179.01 (16)
N1—C1—C6—C5	−174.97 (10)	C13—C14—C15—C16	1.9 (2)
C2—C1—C6—C8	−178.10 (11)	C14—C15—C16—N2	−1.5 (2)
N1—C1—C6—C8	3.78 (17)	C10—C9—C17—N2	−179.26 (14)
C4—C5—C6—C1	−2.06 (16)	C10—C9—C17—C13	0.3 (2)
C7—C5—C6—C1	178.02 (10)	C14—C13—C17—N2	−0.92 (19)
C4—C5—C6—C8	179.13 (11)	C12—C13—C17—N2	179.37 (13)
C7—C5—C6—C8	−0.78 (16)	C14—C13—C17—C9	179.54 (14)
C4—C5—C7—O2	−153.38 (13)	C12—C13—C17—C9	−0.2 (2)
C6—C5—C7—O2	26.54 (18)	C2—C1—N1—O6	−173.03 (12)
C4—C5—C7—O1	27.15 (16)	C6—C1—N1—O6	5.20 (17)
C6—C5—C7—O1	−152.93 (11)	C2—C1—N1—O5	7.53 (17)
C1—C6—C8—O4	−100.90 (14)	C6—C1—N1—O5	−174.24 (12)
C5—C6—C8—O4	77.82 (14)	C15—C16—N2—C17	−0.1 (2)
C1—C6—C8—O3	82.81 (15)	C9—C17—N2—C16	−179.12 (13)
C5—C6—C8—O3	−98.48 (13)	C13—C17—N2—C16	1.3 (2)

### Hydrogen-bond geometry (Å, º)

**Table:**

*D*—H···*A*	*D*—H	H···*A*	*D*···*A*	*D*—H···*A*
O7—H7*A*···O4	0.83 (1)	1.92 (1)	2.7416 (14)	171 (19)
O7—H7*B*···O4^i^	0.84 (1)	2.02 (1)	2.8459 (14)	169 (18)
O1—H1*A*···O7^ii^	0.84 (1)	1.75 (1)	2.5818 (14)	173 (2)
N2—H2*A*···O3^iii^	0.91 (1)	1.74 (1)	2.6425 (14)	176 (18)
C16—H16···O4^iii^	0.93	2.49	3.1166 (17)	125
C16—H16···O2^iv^	0.93	2.39	3.1278 (17)	136
C12—H12···N1	0.93	2.61	3.4866 (19)	157

Symmetry codes: (i) *x*, −*y*−1/2, *z*−1/2; (ii) *x*, *y*, *z*+1; (iii) −*x*+2, *y*+1/2, −*z*+1/2; (iv) −*x*+2, −*y*, −*z*+1.
