# Supplementary figures and images for: Crystal structure of quinolinium 2-carboxy-6-nitro­benzoate monohydrate
Source: Acta Crystallogr E Crystallogr Commun. 2015 Apr 2;71(Pt 5):o270–1. doi: 10.1107/S2056989015006052 (PMC4420104; doi:10.1107/S2056989015006052)

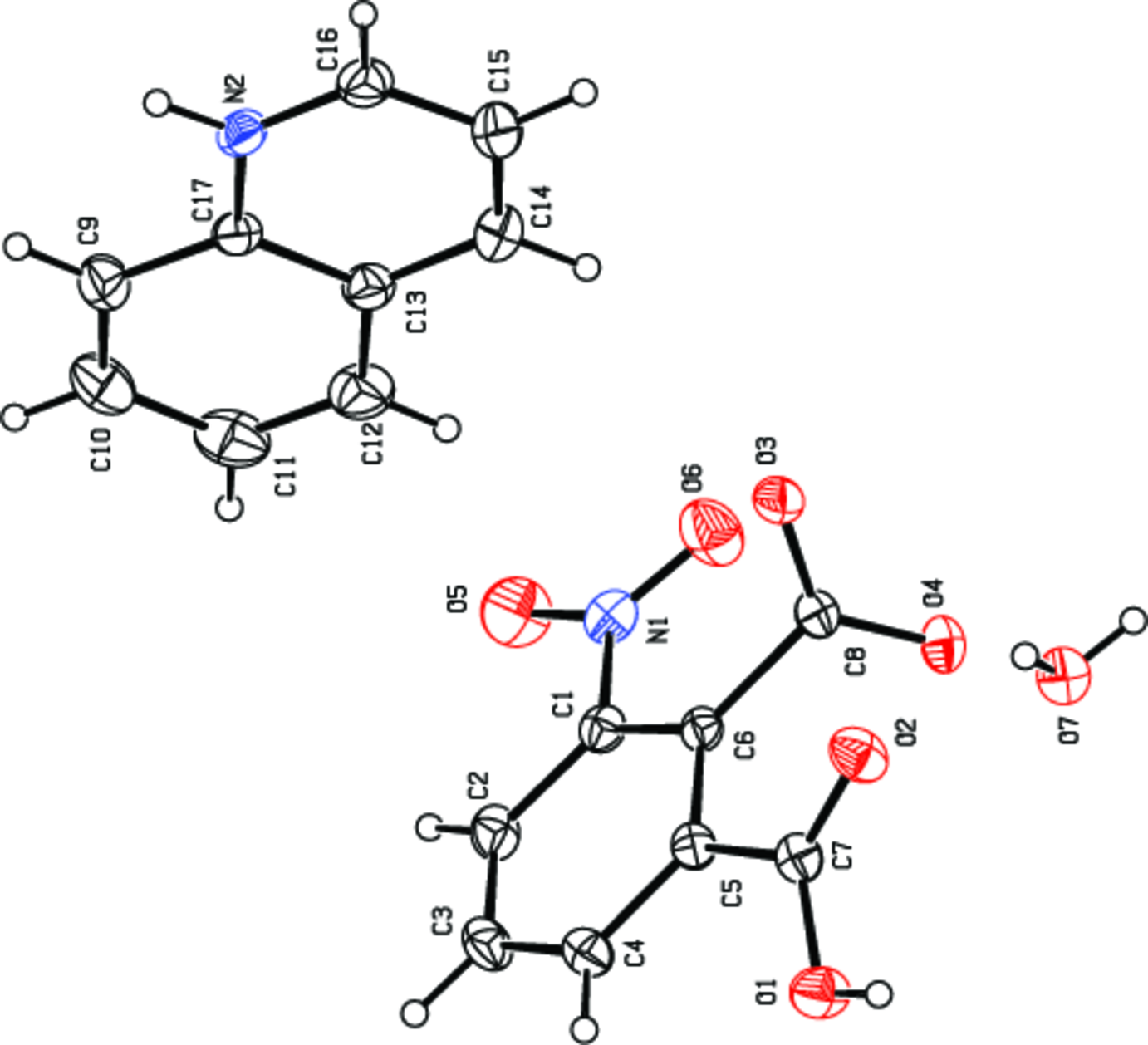

Supplement: Supplementary file 4 [file e-71-0o270-fig1.tif]

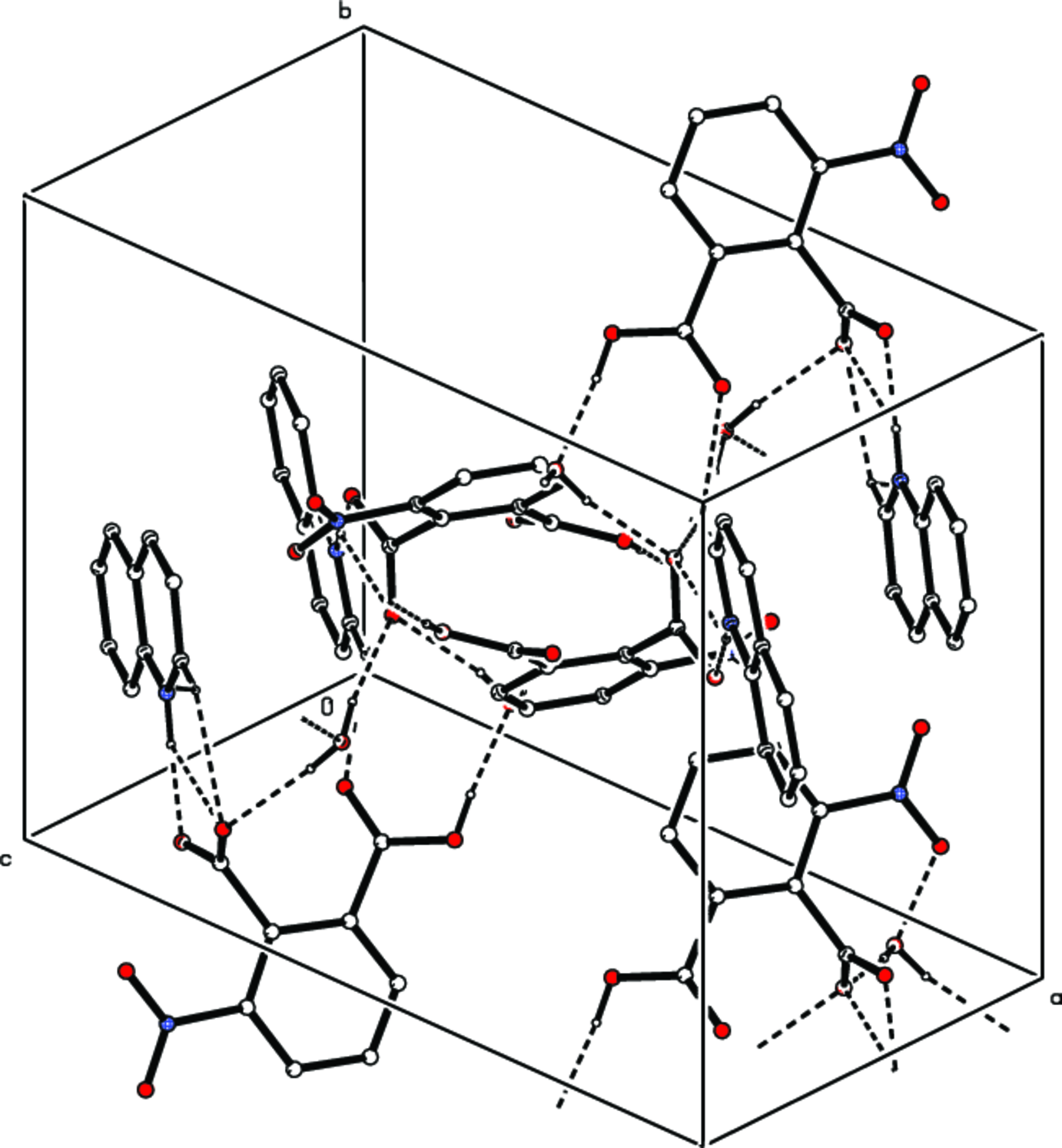

Supplement: Supplementary file 5 [file e-71-0o270-fig2.tif]
